# Fast Analysis Method for Far-Field Scattering Characteristics of Coding Metasurfaces Based on the Equivalent Source Principle

**DOI:** 10.3390/ma19153203

**Published:** 2026-07-27

**Authors:** Rui Yang, Hanzhang Tao, Ge Fan, Junyan Dai, Qiang Cheng

**Affiliations:** 1 State Key Laboratory of Millimeter Waves, Southeast University, Nanjing 210096, China; yangruicqsf@163.com (R.Y.); gefan@seu.edu.cn (G.F.); qiangcheng@seu.edu.cn (Q.C.); 2 State Key Laboratory of Terahertz and Millimeter Waves, Department of Electrical Engineering, City University of Hong Kong, Hong Kong 999077, China; hanzhatao2-c@my.cityu.edu.hk

**Keywords:** coding metasurface, far-field analysis, equivalent source principle, far-field synthesis method

## Abstract

Coding metasurfaces consist of multiple elements with distinct phase (or amplitude) responses. By manipulating the spatial arrangement of these elements, electromagnetic (EM) waves can be flexibly manipulated to achieve various functionalities. To efficiently and accurately calculate the far-field scattering characteristics of coding metasurfaces, some studies have proposed a far-field calculation method based on the equivalent source principle. This method reconstructs the far-field scattering pattern of the metasurface using the total radiation fields generated by the equivalent sources of its elements in space. On this basis, this paper proposes an improved equivalent source method featuring a novel extraction model. Compared with existing methods, it effectively enhances computational accuracy while maintaining high computational efficiency. Comparative analyses against traditional far-field synthesis methods, existing equivalent source methods and full-wave simulation methods, together with experimental measurements, fully verify its excellent performance in both computational efficiency and accuracy.

## 1. Introduction

In recent years, coding metasurfaces have attracted widespread attention due to their powerful capability in manipulating electromagnetic waves, and they have been successfully applied in various fields such as stealth technology [1,2,3,4,5,6,7], beamforming and scanning [8,9,10,11,12,13,14], communications [15,16,17,18,19,20,21], Holographic Imaging [22] and signal control [23]. However, with the continuous expansion of application areas, an increasing number of challenges have emerged. A prominent example is how to rapidly calculate the scattering far-field of large-scale coding metasurfaces with complex structures. Typically, numerical simulation techniques such as the Method of Moments (MoM) [24], Finite-Difference Time-Domain (FDTD) [25,26,27], and Finite Element Method (FEM) [28,29] are employed to simulate the entire coding metasurface for analyzing its scattering characteristics. Although these methods can accurately compute the scattering fields, their computational cost increases drastically as the size and complexity of the metasurface grow. Therefore, achieving rapid calculation of the scattering far-field for large-scale coding metasurfaces has become an urgent problem to be solved.

To address this issue, two main approaches have been proposed for predicting the far-field patterns of coding metasurfaces. One is the traditional far-field synthesis method based on the radiation pattern multiplication (RPM) principle, hereafter referred to as the RPM method. The other is the equivalent source method. Specifically, the RPM method first obtains the far-field pattern of a single coding element through full-wave simulation, and then calculates the overall far-field pattern of the metasurface array according to the principle of pattern multiplication [1,2,3,14]. However, when acquiring the element patterns, this method typically performs independent simulations on isolated elements under open boundary conditions. Consequently, it fails to account for the mutual coupling effects within the array environment, leading to insufficient computational accuracy.

In the equivalent source method, the contribution of each element in the metasurface to the scattering far-field is replaced by its equivalent sources. Early equivalent source methods typically adopted the ‘isolated-element periodic method’ to extract the equivalent sources of each element [30]. This process involves extracting the equivalent electric and magnetic currents from the surface of a single element under periodic boundary conditions. By constructing an infinite periodic model to simulate the interactions between elements, this method accounts for the electromagnetic (EM) coupling between adjacent elements to a certain extent. However, coding metasurfaces are typically aperiodic arrays composed of multiple elements with distinct structural differences. The ideal assumption of the infinite periodic array model deviates significantly from the actual EM environment, resulting in certain errors between the extracted equivalent sources and the true responses of the elements.

To further improve computational accuracy, researchers have incorporated the actual neighboring elements of the target element into the equivalent source extraction model. The ‘Surrounded-element’ method effectively introduces local mutual coupling effects by constructing an extraction model that includes both the target element and its nearest neighbors [31]. However, the limitation of this method is that its extraction model is only applicable to interior elements within an array, neglecting the scenario where elements are located at the edges. Subsequently, by combining the ‘surrounded-element’ concept with periodic boundaries, researchers proposed the extended local periodicity (ELP) method [32]. This method designs independent equivalent source extraction models for elements at different positions, such as interior and edge regions. Nevertheless, this significantly increases the complexity of modeling.

This paper proposes an improved equivalent source method, namely the unified model (UM) method. While accounting for the coupling effects of adjacent elements, this method utilizes a unified model to simultaneously extract the equivalent sources of elements at different positions. It effectively overcomes the limitations of existing methods, which either neglect edge effects or suffer from increased complexity due to the need for constructing independent extraction models for elements at different positions. Furthermore, by adopting open boundary conditions, the proposed method successfully avoids the significant errors observed in the ELP method near resonance frequencies. Comparative studies with the traditional RPM method, the ELP method, and full-wave simulations demonstrate that the proposed method achieves high computational accuracy while maintaining high efficiency. Finally, the experimental results further validate the accuracy and reliability of this method in far-field prediction.

## 2. Fast Far-Field Analysis of Coding Metasurfaces Based on the Equivalent Source Principle

**Figure 1:**
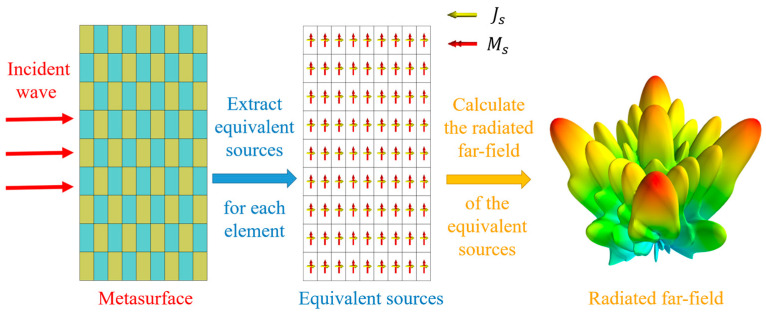
Flowchart of far-field calculation for coding metasurfaces based on the equivalent source method.

According to the equivalent source principle, the contribution of each element in the metasurface to the scattering far-field can be replaced by the equivalent electric current source $J_{s}$ and magnetic current source $M_{s}$ on its enclosing equivalent surface. As shown in Figure 1, when the incident wave illuminates the N × M metasurface, the equivalent sources on the equivalent surface of each element are extracted. Subsequently, the scattering field of the entire metasurface can be characterized by the radiation far-field generated by these equivalent sources in free space. The equivalent sources are obtained as follows:

(1a) $$J_{s}=\hat{n}\times H,$$

(1b) $$M_{s}=-\hat{n}\times E,$$

where E and H denote the electric fields and magnetic fields on the equivalent surface, respectively, and $\hat{n}$ represents the outward unit normal vector of the equivalent surface. The electric field generated by these equivalent current sources $J_{s}$ and magnetic current sources $M_{s}$ in free space can be expressed as follows:

(2) $$E\left(r\right)=-\int_{s}\left[j\omega \mu J_{s}\left(r^{\prime }\right)+\nabla^{\prime }\times M_{s}\left(r^{\prime }\right)\right]∙{\bar{\bar{G}}}_{0}\left(r,r^{\prime }\right)dS^{\prime }.$$

Here, $r^{\prime }$ and r denote the coordinates of the equivalent electric/magnetic current sources and the spatial field point, respectively. ${\overset{̿}{G}}_{0}\left(r,r^{\prime }\right)$ represents the dyadic Green’s function, given by

(3) $${\bar{\bar{G}}}_{0}\left(r,r^{\prime }\right)=\left(\bar{\bar{I}}+\frac{1}{k^{2}}\nabla \nabla \right)\frac{e^{-jk\left|r-r^{\prime }\right|}}{4\pi \left|r-r^{\prime }\right|}.$$

**Figure 2:**
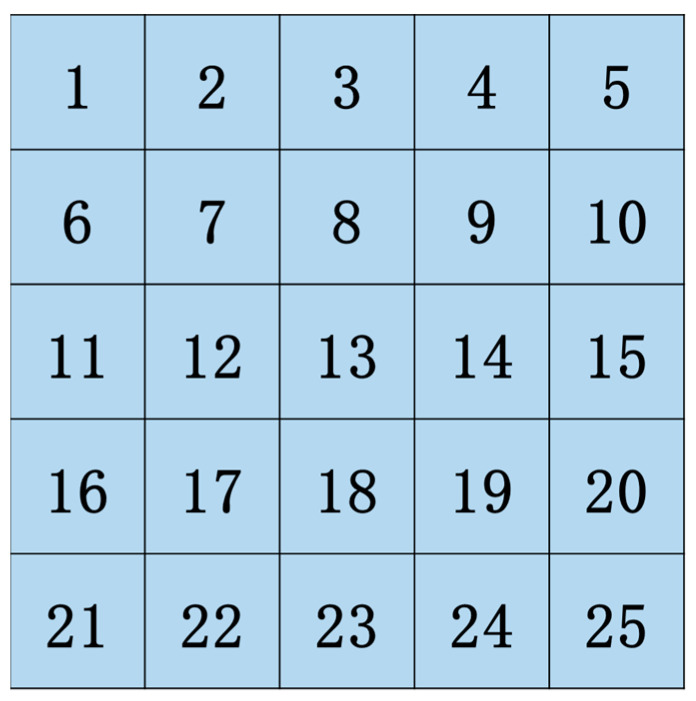
Schematic of the 5 × 5 metasurface (numbers 1 to 25 represent element numbering).

In the proposed UM method, the extraction procedure for $J_{s}$ or $M_{s}$ is as follows: Taking the 5 × 5 (N = M = 5) metasurface shown in Figure 2 as an example, it consists of a total of 25 elements. The positions of these elements can be categorized into 9 distinct types, including four corner regions, four edge regions, and the interior region. Table 1 summarizes the specific regions to which each element of the metasurface belongs.

**Figure 3:**
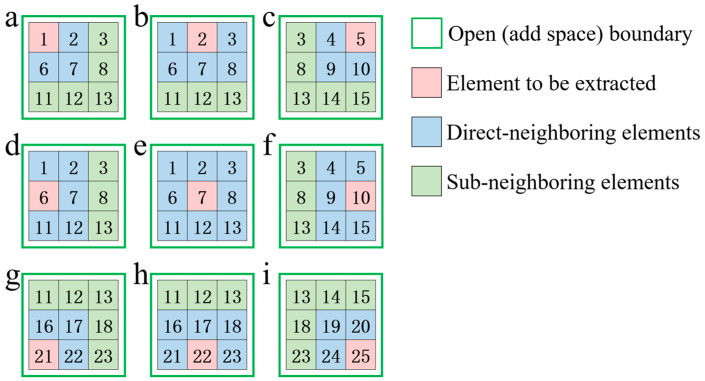
Schematic of the equivalent source extraction models for elements in different regions of the metasurface: (**a**) top-left corner; (**b**) top edge; (**c**) top-right corner; (**d**) left edge; (**e**) interior; (**f**) right edge; (**g**) bottom-left corner; (**h**) bottom edge; (**i**) bottom-right corner.

Since the coupling effect from distant metasurface elements on the target element is weak, only the coupling influence generated by partial neighboring elements is considered. Figure 3a–i illustrate the equivalent source extraction models for the elements in different regions of the metasurface, respectively. When the element to be extracted is located in the interior region of the metasurface (taking element 7 as an example), only the coupling influence from its 8 direct-neighboring elements is considered, as shown in Figure 3e. Therefore, its equivalent source extraction model is a 3 × 3 sub-array centered on it. To maintain consistency, the equivalent source extraction for elements located in the corner and edge regions considers not only the directly adjacent elements but also several sub-neighboring elements. Specifically, when the element to be extracted is located in the top, left, right, and bottom edge regions (taking elements 2, 6, 10, and 22 as examples, respectively), the equivalent source extraction model is a 3 × 3 sub-array composed of the target element, its direct-neighboring elements, and some sub-neighboring elements, as shown in Figure 3b, Figure 3d, Figure 3f, and Figure 3h, respectively. Similarly, when the element to be extracted is located in the top-left, top-right, bottom-left, and bottom-right corner regions, the extraction model includes not only the target element (elements 1, 5, 21, and 25) but also its direct-neighboring and sub-neighboring elements, as illustrated in Figure 3a,c,g,i.

Since all the extraction models are 3 × 3 sub-arrays, the equivalent sources of multiple elements can be extracted through a single simulation when their sub-array encodings are identical. As shown in Figure 3a,b,d,e, although the positions of the elements to be extracted differ, their equivalent source extraction models are exactly the same. Therefore, the equivalent sources for elements 1, 2, 6, and 7 can be simultaneously extracted with just one simulation. Consequently, the proposed extraction approach is applicable to elements located at the edges and corners of the metasurface and significantly reduces the number of required simulations.

**Figure 4:**
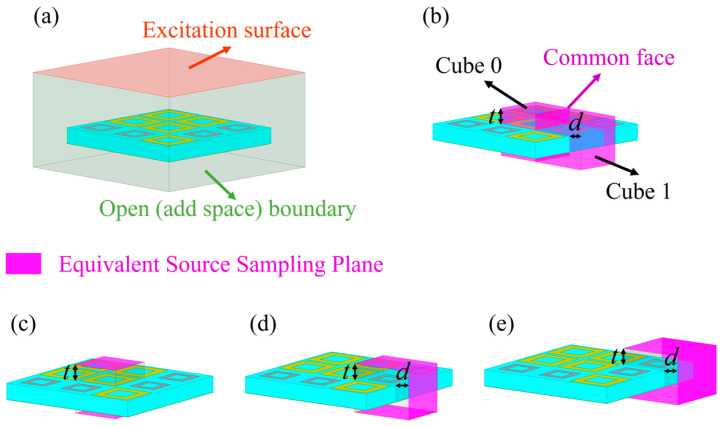
(**a**) Schematic of the simulation setup for equivalent source extraction; (**b**) schematic of the closed equivalent cube enclosing the element; equivalent source sampling planes for an element in the (**c**) interior, (**d**) edge and (**e**) corner region.

The entire 3 × 3 sub-array is simulated under normal incidence, and the boundary conditions on the four lateral sides and the bottom of the sub-array are uniformly set to “Open (add space)”, as shown in Figure 4a. According to the equivalent source principle, the scattered far-field contribution of a metasurface element can be represented by equivalent sources distributed on a closed surface enclosing the element. Theoretically, the equivalent surface is composed of the six faces of a closed cube, irrespective of whether the element is located in the interior, edge, or corner region of the metasurface. Figure 4b illustrates the equivalent cubes, Cube 0 and Cube 1, enclosing the interior element and the right-edge element, respectively. As shown in the figure, the right face of Cube 0 and the left face of Cube 1 coincide, but they have opposite outward normal directions. Based on Equations (1a) and (1b), the equivalent sources on these two faces are identical in magnitude but opposite in direction, thus canceling each other out. Similarly, as long as an element has adjacent elements in a certain direction, the equivalent sources on the corresponding face in that direction can be neglected. It should be noted that this omission does not imply that the equivalent surface is physically open. The theoretical equivalent surface for each element remains a closed cube; the sources on the shared faces are omitted only because their contributions can be eliminated.

Consequently, the equivalent source extraction strategy is formulated as follows. For elements located in the interior region of the metasurface, equivalent sources are extracted only from the top and bottom surfaces, with the sampling planes placed at a distance of t from the element, as shown in Figure 4c. For elements located at the edges or corners, the outer lateral faces are additionally included in the extraction process, as illustrated in Figure 4d and Figure 4e, respectively. To ensure accuracy, a spacing of d is maintained between the outer sampling planes and the element. Meanwhile, the top and bottom sampling planes are correspondingly extended outward by a distance of d.

### 2.1. Methodological Innovations of the UM Method

**Figure 5:**
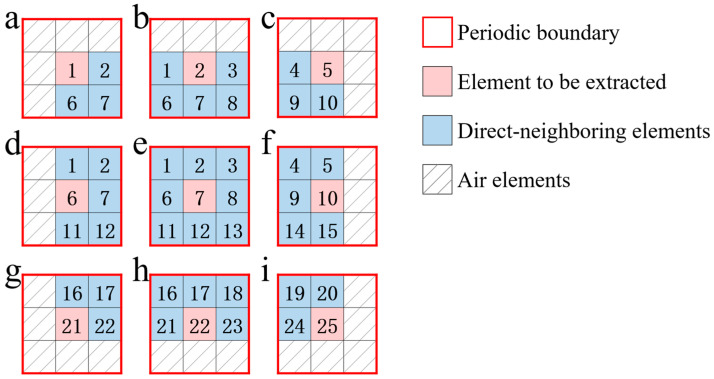
Equivalent source extraction models for elements in different regions of the ELP method: (**a**) top-left corner; (**b**) top edge; (**c**) top-right corner; (**d**) left edge; (**e**) interior; (**f**) right edge; (**g**) bottom-left corner; (**h**) bottom edge; (**i**) bottom-right corner.

Existing equivalent source methods have certain limitations in dealing with elements located at different regions of a metasurface. The “surrounded element” method is applicable only to interior elements [31] and does not consider edge effects. In contrast, the ELP method [32] takes edge elements into account and establishes separate extraction models for different regions, as illustrated in Figure 5.

Figure 5a–i show the extraction models for target elements located in the top-left corner, top edge, top-right corner, left edge, interior, right edge, bottom-left corner, bottom edge, and bottom-right corner regions, respectively. As shown in Figure 5e, when the target element (e.g., element 7) is located in the interior region, the extraction model consists of a 3 × 3 sub-array formed by the target element and its eight directly adjacent elements. For elements located at the edges or corners of the metasurface, dummy air elements are introduced to fill the vacant neighboring positions. In this way, the target element remains at the center of the extraction model, as shown in Figure 5a–d,f–i.

However, this extraction strategy incurs high computational costs. As shown in Figure 5a,b,d,e, four separate modeling simulations are required with the conventional ELP method to extract the equivalent sources of elements 1, 2, 6 and 7. In contrast, as previously mentioned, the proposed UM method requires only one, significantly reducing the modeling and simulation workload during the equivalent source extraction phase. Furthermore, the ELP method employs periodic boundary conditions while considering neighboring elements, leading to a significant degradation in far-field calculation accuracy at the resonant frequency [33].

In summary, the proposed UM method exhibits the following methodological innovations:

1. A more comprehensive extraction strategy: Compared with the “surrounded element” method, the UM method comprehensively considers the scenarios where the target element is located in different regions of the metasurface.
2. Lower complexity in the equivalent source extraction phase and higher computational accuracy: Compared with the ELP method, the UM method can extract the equivalent sources for elements in any region using a unified model, significantly reducing the complexity during this process. Furthermore, the UM method employs “Open (add space)” boundary conditions, effectively avoiding the large errors at resonant frequencies in the ELP method.

### 2.2. The Influence of Sampling Parameters on Accuracy

**Figure 6:**
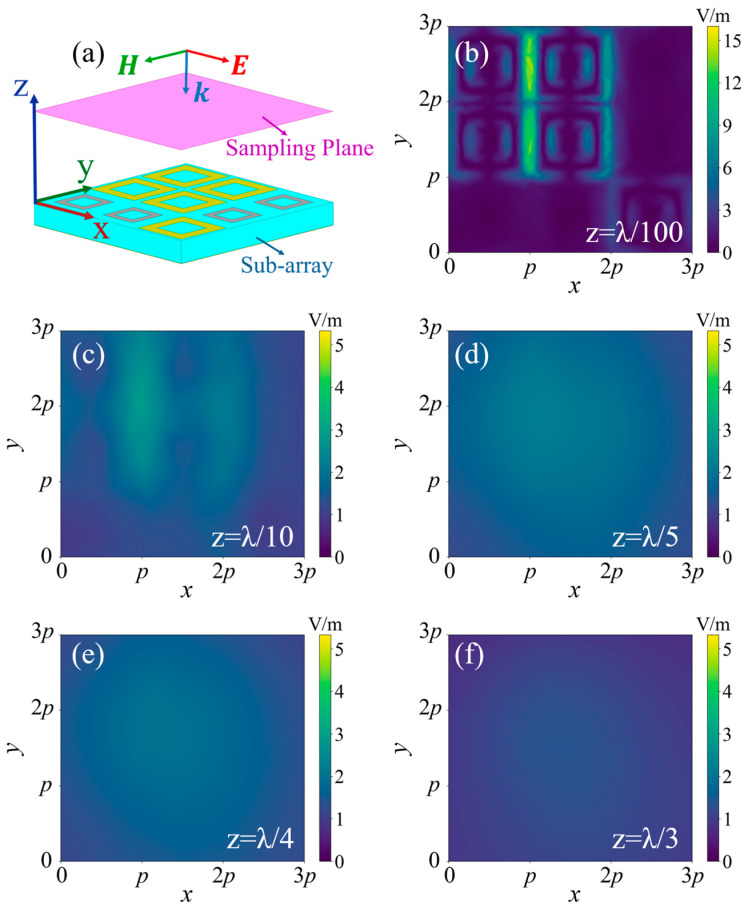
(**a**) Schematic of the sub-array and the sampling plane; electric field intensity distributions at (**b**) *z* = λ/100, (**c**) *z* = λ/10, (**d**) *z* = λ/5, (**e**) *z* = λ/4, and (**f**) *z* = λ/3. Here, *p* denotes the element period.

Since the EM current distribution near the metasurface varies drastically, two key parameters must be carefully set to ensure that the extracted equivalent sources can accurately reconstruct the scattering characteristics of the actual elements: the distances *t* and *d* between the equivalent source extraction surface and the metasurface, as well as the step size *l* between adjacent sampling points. To clarify the relationship between the electric field variation rate and the sampling distance, this paper utilizes the frequency-domain solver in the CST Microwave Studio 2020. The electric field generated by the 3 × 3 sub-array shown in Figure 6a under normal incidence of an *x*-polarized plane wave is simulated, and the electric field intensity distributions on the sampling planes at various distances directly above the metasurface are observed. The specific geometric parameters of the elements in the sub-array are provided in Section 3.1.

**Figure 7:**
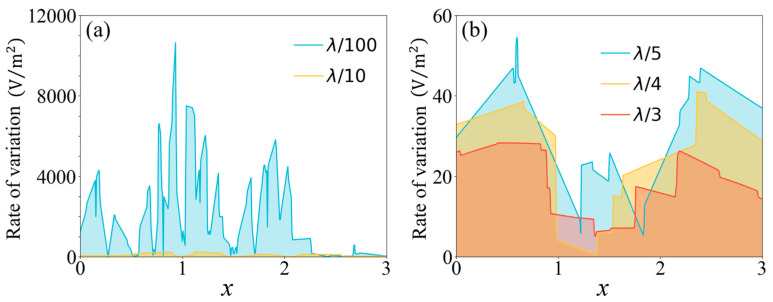
Variation rate of the $E_{x}$ component along the *x*-direction at *y* = 3*p*/2 for different sampling distances: (**a**) *z* = λ/100 and *z* = λ/10; (**b**) *z* = λ/5, *z* = λ/4, and *z* = λ/3.

As shown in Figure 6b–f, when the sampling plane is placed very close to the sub-array (*z* = λ/100), the electric field exhibits drastic fluctuations. As the sampling distance gradually increases, the variation trend of the electric field becomes smoother. To intuitively illustrate this phenomenon, this paper calculates the variation rate of the electric field $E_{x}$ component along the *x*-direction on the line *y* = 3*p*/2 of each sampling plane. It can be seen from Figure 7a that as the sampling distance increases from λ/100 to λ/10, the electric field variation rate drops rapidly. However, the maximum value at this stage still reaches approximately 250, suggesting that substantial fluctuations remain. When the distance is further increased to λ/5, the maximum variation rate decreases to around 55. As the distance reaches λ/4 and λ/3, the variation rate curves basically stabilize. Although the peak values continue to decrease slightly, the change is no longer significant. In some regions, the variation rate at λ/4 is even lower than that at λ/3, as shown in Figure 7b.

The above results indicate that when the distance between the sampling plane and the sub-array reaches λ/4 or greater, the spatial variation in the EM field tends to become smooth. Therefore, selecting *t* ≥ λ/4 and *d* ≥ λ/4 as the equivalent source extraction locations for the top/bottom surfaces and the side surfaces of the target element, respectively, can effectively avoid the risk of undersampling caused by drastic EM field fluctuations.

**Figure 8:**
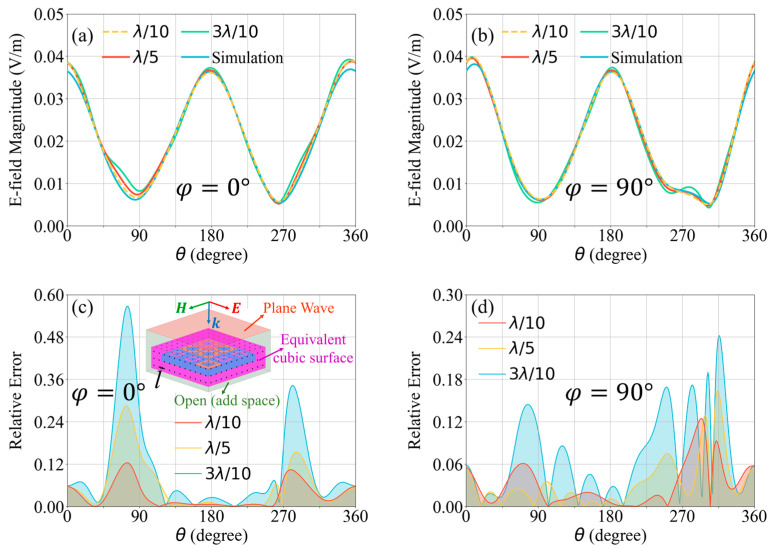
Comparison between equivalent source calculation results and full-wave simulation results under different sampling step sizes: (**a**) *φ* = 0°; (**b**) *φ* = 90°; (**c**) relative error at *φ* = 0°; (**d**) relative error at *φ* = 90°.

After determining the appropriate sampling distance, this paper further investigates the impact of the sampling step size *l* on the accuracy of the reconstructed field. As shown in the inset of Figure 8c, the equivalent surface is set as a cubic surface that completely encloses the sub-array while maintaining an appropriate gap with it. After obtaining the electric field E and magnetic field H at each sampling point on the equivalent surface through full-wave simulation, the electric field distribution generated by the sub-array in free space can be calculated based on Equations (1a), (1b), and (2).

Figure 8a,b present the scattering electric fields calculated by equivalent sources and the full-wave simulation results on the *φ* = 0° and *φ* = 90° cuts, respectively, under different sampling step sizes. It can be seen that when the sampling step size is λ/10, the results obtained from equivalent sources show excellent agreement with the full-wave simulation. However, as the sampling step size increases, the calculation accuracy degrades. As shown in Figure 8c,d, when the sampling step size is 3λ/10, the maximum relative error between the calculated and simulated results exceeds 56%. The maximum relative error is approximately 28% for a step size of λ/5, while it is only about 12% for λ/10. Therefore, to ensure the reconstruction accuracy of the metasurface scattering far-field, the sampling step size should not exceed λ/10.

In consideration of mutual coupling between adjacent elements, the UM method proposed in this section utilizes a unified 3 × 3 sub-array model to extract equivalent sources for all the elements. Furthermore, the appropriate extraction distance and sampling step size are investigated. Additionally, in Supplementary Material S1, a sub-array composed of the Jerusalem-cross elements described in Section 3.2 is used as an example to further verify that the above equivalent-source extraction settings are insensitive to the element structure and exhibit good generality. In the next section, the performance of this method in terms of computational efficiency and accuracy will be verified through comparative analyses with the RPM method, the ELP method, and the full-wave simulation results.

## 3. Simulation Validation

To verify the effectiveness of the proposed UM method, this section takes a 1-bit chessboard-like coding metasurface and a beam-steering metasurface as examples. The scattering far-fields are calculated using the UM method, ELP method, RPM method, and full-wave simulation, respectively, followed by a comprehensive analysis of their performance in terms of computational accuracy and efficiency.

### 3.1. Chessboard-like Coding Metasurface

**Figure 9:**
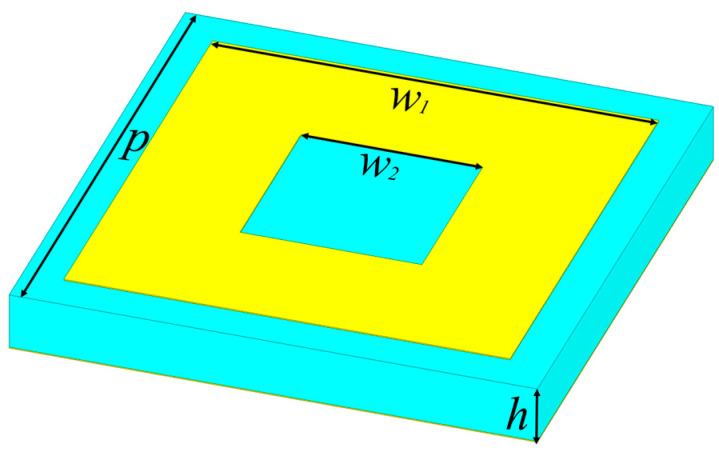
Schematic of the square ring element.

In this section, a 1-bit chessboard-like coding metasurface is taken as a case study. The elements of the metasurface adopt a conventional ‘metal–dielectric–metal’ structure, where a metallic square ring patch is placed on top of the dielectric substrate, and a metallic ground plane is located at the bottom, as shown in Figure 9. The element’s period is *p* = 15 mm. The dielectric substrate has a thickness of *h* = 1 mm, a relative permittivity of $\varepsilon_{r}=2.65$, and a loss tangent of tanδ=0.0015. Both the metallic patches and the ground plane have a thickness of 0.035 mm. The specific dimensions of the square ring patches for the two coding elements are as follows: for the element “0”, w1 = 13.1 mm and w2 = 7.5 mm; for the element “1”, w1 = 9 mm and w2 = 5.5 mm.

**Figure 10:**
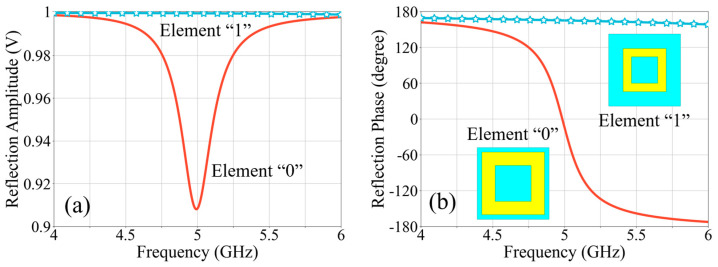
Reflection characteristics of the “0” and “1” elements: (**a**) reflection amplitude; (**b**) reflection phase.

**Figure 11:**
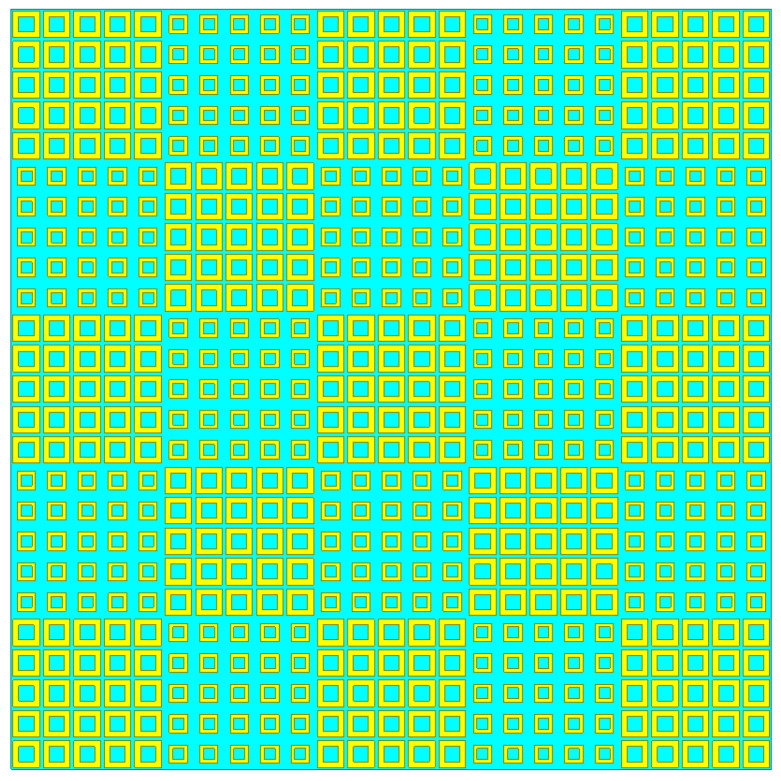
The chessboard metasurface with square ring elements.

Figure 10a,b present the reflection magnitude and phase of the two elements under normal incidence, respectively. As shown in the figures, both elements exhibit low-loss characteristics around 5 GHz, with a reflection phase difference of approximately 180°, which fully satisfies the performance requirements for 1-bit coding elements. The arrangement of the metasurface is as follows: identical elements are first arranged into a 5 × 5 supercell, and these supercells are then organized into a 25 × 25 chessboard-like metasurface following the “01010/10101/01010/10101/01010” pattern, as shown in Figure 11.

The scattering far-fields of the metasurface are calculated using full-wave simulation, the UM method, the ELP method, and the RPM method, respectively. All four methods rely on full-wave simulation technology: the full-wave simulation method directly simulates the entire metasurface to solve for its scattering far-field; the UM and ELP methods require simulations to extract the equivalent sources of the elements; while the RPM method requires simulations to obtain the far-field pattern of the “0” and “1” elements. All the aforementioned simulations are performed using the frequency domain solver in CST Microwave Studio 2020. Specifically, the ELP method adopts periodic boundary conditions and is excited by Floquet ports; the other three methods employ plane wave excitation with boundary conditions set to “Open (add space)”. In addition, the polarization direction of the incident waves for all four methods is set to *x*-polarization.

As mentioned in Section 2, in the UM method, the equivalent sources can be extracted via a 3 × 3 sub-array model regardless of whether the element is located at a corner, edge, or interior region of the metasurface. The chessboard metasurface studied in this section can be divided into 23 × 23 sub-arrays, comprising a total of 18 coding patterns. Therefore, the UM method only requires extracting equivalent sources for all the elements within these 18 sub-arrays to obtain the data needed for reconstructing the scattering far-field of the entire metasurface. In contrast, the ELP method is more sensitive to the position of the elements, requiring dedicated extraction models specifically for corner and edge elements. Consequently, in addition to the aforementioned 18 3 × 3 sub-arrays, 28 special-region models must be constructed. These include 4 corner models and 24 edge models (6 for each of the top, bottom, left, and right edges). Additionally, since the ELP method employs periodic boundary conditions and Floquet ports, the boundary constraints and port-mode treatments are more complicated. As a result, the simulation time for a single sub-array model is approximately 4.3 min, which is slightly longer than that of the UM method, approximately 2.6 min. Consequently, the total time required for equivalent source extraction using the ELP method is about 3.3 h, which is much longer than that required by the UM method, about 47 min.

For the equivalent source extraction model in the UM method, the key parameters are set as follows: The sampling step size *l* is set to λ/12 (5 mm), and the distances from the top/bottom surfaces and side surfaces of the equivalent cube to the element are set to *t* = 17 mm and *d* = 15 mm, respectively. For the ELP method, the sampling step size is also set to 5 mm, with the equivalent source sampling plane located 17 mm directly above the element to be extracted.

**Figure 12:**
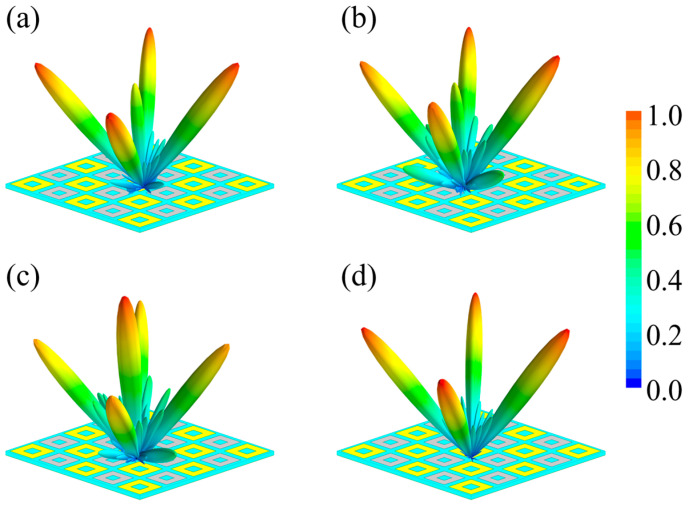
Normalized far-field patterns of the metasurface calculated by (**a**) full-wave simulation; (**b**) UM method; (**c**) ELP method; (**d**) RPM method.

Figure 12a–d illustrate the normalized far-field patterns of the metasurface calculated by the full-wave simulation, UM, ELP, and RPM methods, respectively. As shown in the figures, the result obtained by the UM method is in excellent agreement with that of the full-wave simulation. It not only accurately reproduces the four scattering beams distributed around the perimeters but also precisely captures the low-amplitude scattering beam located in the central region. Although the ELP method successfully recovers the central beam, its amplitude is significantly larger than that of the surrounding beams, resulting in a considerable deviation from the full-wave simulation results. In contrast, the RPM method can only recover the peripheral scattering beams and completely fails to capture the central beam.

**Figure 13:**
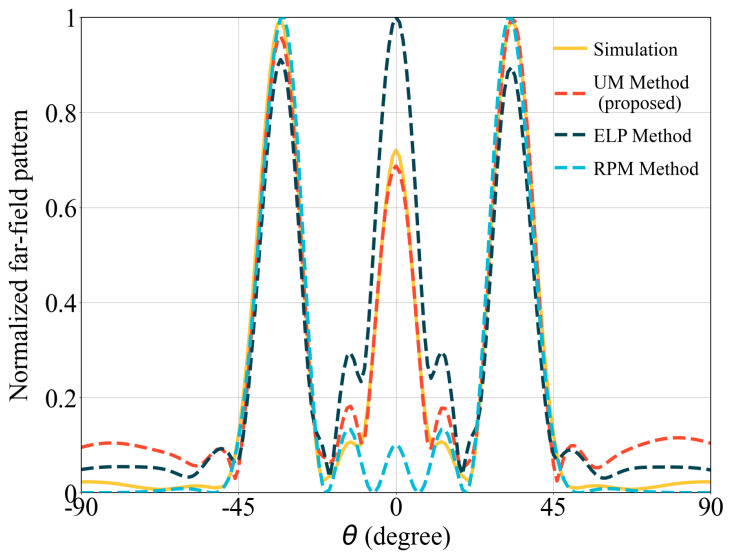
Normalized far-field patterns of the metasurface in the *φ* = 45° plane.

To more clearly illustrate the differences among the results of various methods, Figure 13 presents the normalized far-field patterns in the *φ* = 45° plane. As shown in Figure 13, although the RPM method is in excellent agreement with the full-wave simulation at *θ* = ±33°, it completely fails to capture the central beam at *θ* = 0°. While the ELP method successfully recovers the central beam at *θ* = 0°, its amplitude is significantly larger than that of the surrounding scattering beams, exhibiting a considerable deviation from the full-wave simulation results. In contrast, the results obtained by the proposed UM method are in excellent agreement with those of the full-wave simulation, showing only minor discrepancies in certain regions.

The above qualitative observations are further supported by the Pearson correlation coefficient and the root mean square error (RMSE), which were used to characterize the consistency in the variation trend and quantify the absolute deviation between the results obtained by different computational methods and the full-wave simulation result, respectively. The Pearson correlation coefficient is defined as

(4) $$r=\frac{\sum_{i=1}^{N}\left(f_{cal,i}-{\bar{f}}_{cal}\right)\left(f_{sim,i}-{\bar{f}}_{sim}\right)}{\sqrt{\sum_{i=1}^{N}{\left(f_{cal,i}-{\bar{f}}_{cal}\right)}^{2}}\sqrt{\sum_{i=1}^{N}{\left(f_{sim,i}-{\bar{f}}_{sim}\right)}^{2}}},$$

and the RMSE is defined as

(5) $$RMSE=\sqrt{\frac{1}{N}\sum_{i=1}^{N}{\left(f_{cal,i}-f_{sim,i}\right)}^{2}}.$$

Here, $f_{cal,\;i}$ and $f_{sim,\;i}$ denote the normalized far-field intensities at the *i*-th sampling point obtained from the calculation method and full-wave simulation, respectively; ${\bar{f}}_{cal}$ and ${\bar{f}}_{sim}$ are their corresponding mean values; and N is the total number of sampling points.

As shown in Table 2, the Pearson correlation coefficient between the result obtained by the UM method and the full-wave simulation result reaches 0.991, while the RMSE is only 0.060. This indicates that the far-field pattern calculated by the UM method is highly consistent with the full-wave simulation result in terms of the overall variation trend and exhibits a relatively small absolute deviation. In contrast, the Pearson correlation coefficient of the ELP method is 0.947, indicating that it can still capture the overall variation trend of the far-field pattern. However, its RMSE increases to 0.105, suggesting a noticeable error in the beam amplitude distribution, which is consistent with the overestimation of the central beam amplitude at *θ* = 0° observed in Figure 13. The Pearson correlation coefficient of the RPM method decreases to 0.893, and the RMSE increases to 0.145, indicating the lowest prediction accuracy among the three methods, mainly because it fails to capture the central beam at *θ* = 0°. Overall, the proposed UM method achieves the best agreement with the full-wave simulation and the highest far-field prediction accuracy among the three methods.

The last column of Table 2 summarizes the computational time of each method under the same hardware configuration (Intel i7-7700 CPU and 16 GB RAM). The full-wave simulation requires approximately 4.5 h, whereas the proposed UM method takes only about 80 s. By extracting only the equivalent currents above the elements while ignoring those on the backside and surrounding surfaces, the ELP method further reduces the computational time to 24 s. The RPM method is the fastest, requiring only 1.6 s. Although the ELP and RPM methods exhibit higher computational efficiency, their calculated results show relatively large deviations from the full-wave simulation. Considering both accuracy and efficiency, the UM method significantly reduces the computational time while maintaining high accuracy, thereby demonstrating superior overall performance.

To investigate the minor discrepancies between the UM method and the full-wave simulation, an in-depth analysis was conducted. The results indicate that these discrepancies primarily stem from the significant size difference between the 3 × 3 equivalent source extraction model and the complete metasurface array. The smaller size of the extraction model exacerbates edge effects, leading to certain deviations between the extracted equivalent sources and those in the full-array case. Furthermore, only the coupling effects of local neighboring elements are considered during the equivalent-source extraction process, while the weak interactions with more distant elements are neglected, which also reduces the accuracy to some extent.

Although incorporating more neighboring elements into the extraction model could theoretically further reduce the error, it would lead to a dramatic increase in the number of coding combinations, thereby significantly increasing the complexity of the equivalent source extraction. In Supplementary Material S2, the equivalent-source extraction model is extended to a 5 × 5 sub-array. The Pearson correlation coefficient increases from 0.991 to 0.998, and the RMSE decreases from 0.060 to 0.016, indicating improved agreement with the full-wave simulation, especially in the sidelobe regions. However, the improvement in the main-beam directions is limited, as the 3 × 3 model already predicts the main beams accurately. Meanwhile, the number of sub-array coding patterns increases to 50, and the simulation time for a single model increases to 3.2 min, resulting in a total equivalent source extraction time of approximately 2.7 h. Therefore, to strike a balance between computational accuracy and problem complexity, adopting a 3 × 3 sub-array model serves as a reasonable and effective trade-off.

### 3.2. Beam-Steering Metasurface

**Figure 14:**
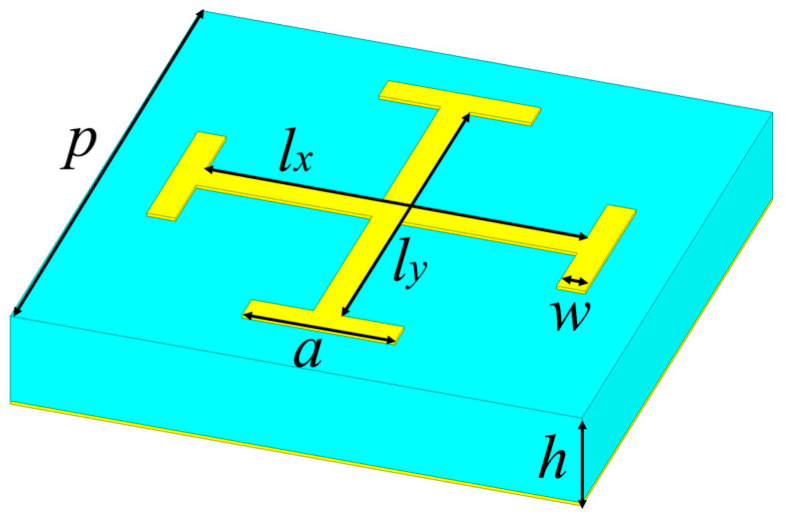
Schematic of the Jerusalem cross element.

To further verify the generality and robustness of the proposed UM method, this subsection presents an analysis of the scattering far-field of a 1-bit beam-steering metasurface composed of Jerusalem cross elements. Figure 14 illustrates the specific structures of the “0” and “1” coding elements of the metasurface. The metallic pattern layer is located on top of the dielectric substrate, while the metallic ground plane is placed at the bottom. The element has a period of *p* = 6 mm and a substrate thickness of *h* = 2 mm. The relative permittivity of the substrate is 2.65, and the loss tangent is 0.001. Both the metallic patch and the ground plane have a thickness of 0.035 mm. By adjusting the arm length and line width of the cross structure in the *x*-direction, two coding elements with a reflection phase difference of approximately 180° under *x*-polarized excitation are designed. The geometric parameters for the “0” element are $l_{x}=2.5\;mm$, $l_{y}=4\;mm$, w=0.18 mm and a=1.6 mm, and those for the “1” element are $l_{x}=4\;mm$, $l_{y}=4\;mm$, w=0.3 mm and a=1.6 mm.

**Figure 15:**
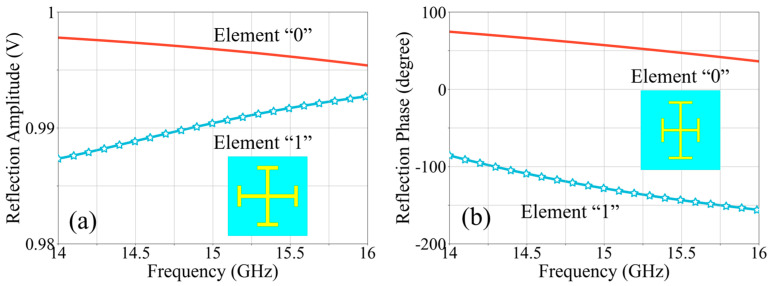
Reflection characteristics of the “0” and “1” Jerusalem cross elements: (**a**) reflection amplitude; (**b**) reflection phase.

**Figure 16:**
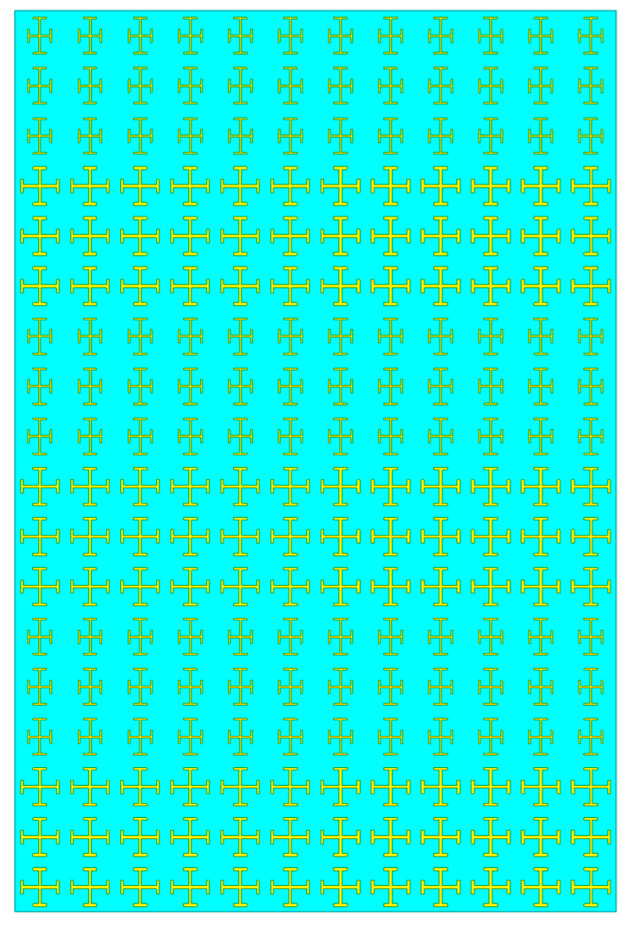
The beam-steering metasurface with the Jerusalem cross element.

**Figure 17:**
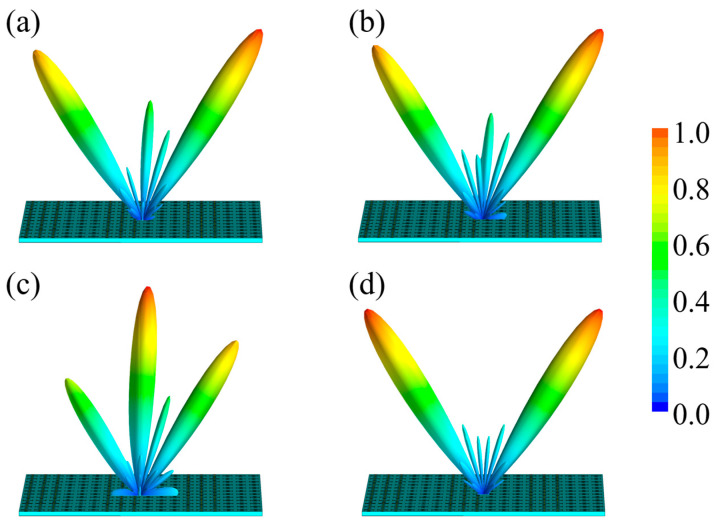
Normalized far-field patterns of the beam-steering metasurface calculated by (**a**) full-wave simulation; (**b**) UM method; (**c**) ELP method; (**d**) RPM method.

Figure 15a,b illustrate the reflection magnitudes and reflection phases of the two coding elements, respectively. As can be seen, the reflection phase difference between the two elements is approximately 180° at 15 GHz, and both exhibit high reflectivity. Using these coding elements, a beam-steering metasurface is designed. As shown in Figure 16, the metasurface consists of 18 × 12 elements. It is divided into groups of three rows, with each group adopting the same element. The coding sequence of the metasurface can be represented as “010101”. At 15 GHz, the normalized far-field patterns of this metasurface are calculated using the full-wave simulation, UM, ELP, and RPM methods, with the corresponding results presented in Figure 17a–d, respectively. In both the UM and the ELP methods, the distance between the equivalent source extraction plane and the target element is set to 6 mm, and the sampling step is set to 2 mm.

**Figure 18:**
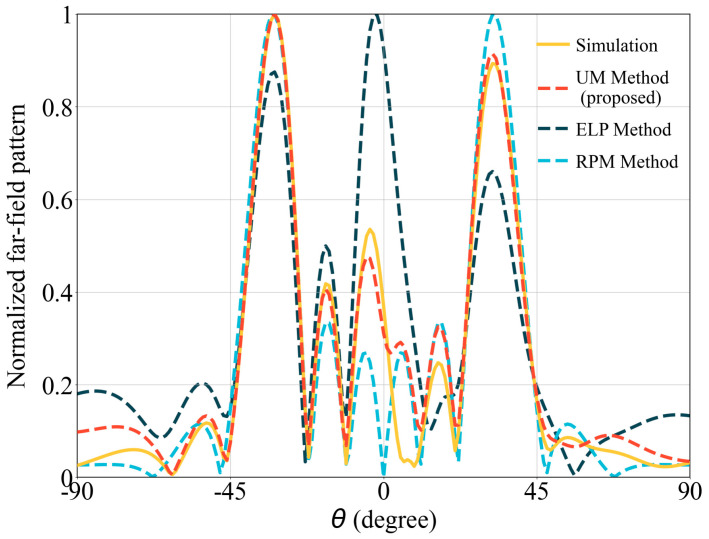
Normalized far-field patterns of the metasurface in the *φ* = 90° plane.

As shown in Figure 17, the result obtained by the UM method agrees well with the full-wave simulation result, whereas the results obtained by the ELP and RPM methods exhibit noticeable deviations. To more clearly compare the differences between the results of each computational method and the full-wave simulation, Figure 18 presents the normalized scattering far-field pattern in the *φ* = 90° plane. According to the full-wave simulation result represented by the yellow solid line in Figure 18, the designed metasurface deflects the reflected beams toward *θ* = ±32°, with the scattering beam at *θ* = 32° exhibiting a slightly larger amplitude. Meanwhile, a weak sidelobe appears near *θ* = 4°. As shown by the red dashed line, the far-field pattern calculated by the UM method is highly consistent with the full-wave simulation result. It accurately reproduces the main scattering beams at *θ* = ±32°, with only slight deviations in the sidelobe region. In contrast, although the ELP method can predict the two reflected beams at *θ* = ±32° and their amplitude difference, it produces an obviously overestimated scattering beam near *θ* = 4°, resulting in a large deviation from the full-wave simulation. The RPM method predicts two scattering beams at *θ* = ±32° with nearly equal amplitudes and fails to reproduce the weak sidelobe near *θ* = 4°, thus still showing a noticeable discrepancy from the full-wave simulation result.

Table 3 summarizes the Pearson correlation coefficient, RMSE, and computational time of each computational method, with the full-wave simulation used as the reference for accuracy evaluation. It can be seen that the Pearson correlation coefficient between the UM method and the full-wave simulation reaches 0.986, while the RMSE is only 0.052, indicating that the UM method achieves the highest prediction accuracy for the far-field pattern. In contrast, the Pearson correlation coefficients of the ELP and RPM methods are 0.814 and 0.955, respectively, and their RMSE values increase to 0.173 and 0.086, respectively, suggesting larger deviations from the full-wave simulation than those of the UM method. In terms of computational efficiency, the full-wave simulation takes approximately 1.2 h, whereas the UM method requires only 36 s, significantly reducing the computational time. Although the computational times of the ELP and RPM methods are further reduced to 11 s and 0.6 s, respectively, their prediction accuracy is lower than that of the UM method, especially for the ELP method.

Overall, this example further demonstrates that the UM method can achieve more accurate far-field prediction while maintaining high computational efficiency. Its effectiveness is less affected by variations in the element structure, coding distribution and operating frequency, indicating its general applicability. According to the discretized form of Equation (2), the UM method needs to calculate the radiation contribution from each equivalent source to each observation direction. Therefore, its computational complexity is $O\left(N_{s}N_{o}\right)$, where $N_{o}$ is the number of observation directions and $N_{s}$ is the number of equivalent source sampling points. When the equivalent source sampling step size on each element is fixed, such as λ/10, $N_{s}$ is approximately proportional to the number of metasurface elements, $N_{cell}$. Therefore, for a fixed number of observation directions, the far-field reconstruction cost of the UM method scales approximately linearly with the metasurface size.

In practical implementation, there is a trade-off between memory requirements and computational speed. Since the storage requirements for the source-point coordinates, observation-direction coordinates, and equivalent source data scale only as $O\left(N_{s}{+N}_{o}\right)$, they are usually negligible compared with the storage requirement of the Green’s function matrix. Therefore, the memory requirement discussed here mainly refers to the storage of the Green’s function matrix. If the complete Green’s function matrix between all source points and observation directions is explicitly stored, the memory requirement is $O\left(N_{s}N_{o}\right)$. For large-scale coding metasurfaces, a block-wise calculation strategy can be adopted, in which only $B_{o}$ observation directions are processed at a time, thereby reducing the storage requirement of the Green’s function matrix to $O\left(N_{s}B_{o}\right)$. This strategy does not change the overall computational complexity of $O\left(N_{s}N_{o}\right)$, but the actual computation time may increase because the Green’s function needs to be generated separately for different blocks.

## 4. Experimental Validation

**Figure 19:**
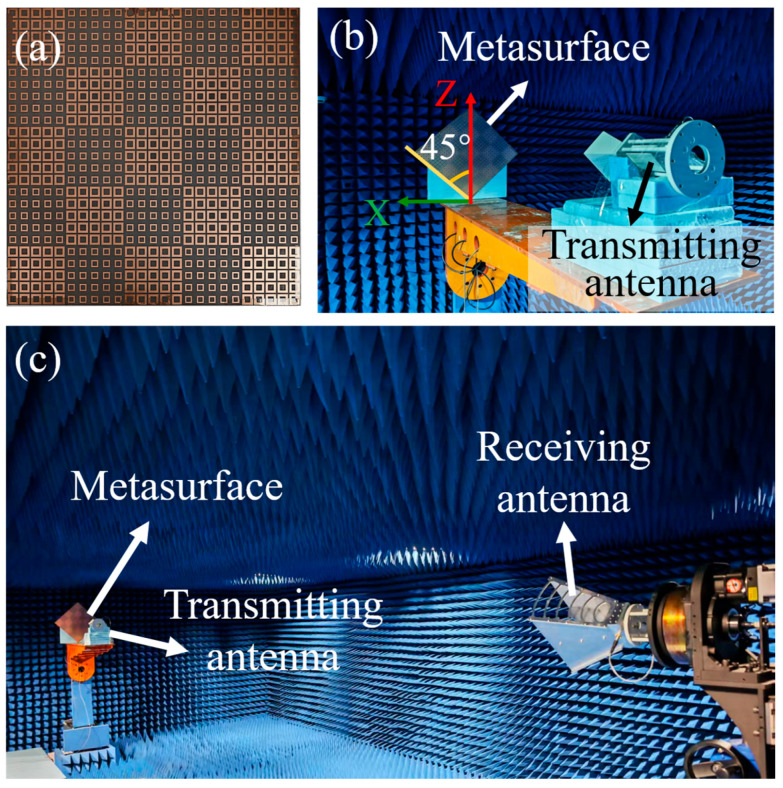
(**a**) Photograph of the metasurface prototype; (**b**) details of the transmitting end in the far-field measurement system; (**c**) normalized far-field pattern measurement system.

In this section, the chessboard metasurface designed in Section 3.1 is used as an example to experimentally verify the effectiveness of the proposed UM method. A prototype of the metasurface was fabricated using printed circuit board (PCB) technology. The overall dimensions of the sample are 375 mm × 375 mm, as shown in Figure 19a. The dielectric substrate is F4B, with a thickness of 1 mm, a relative permittivity of 2.65, and a loss tangent of 0.0015.

The far-field patterns of the metasurface prototype were measured in an anechoic chamber along the *φ* = 45° and *φ* = 135° planes. Figure 19 illustrates the measurement setup using the *φ* = 45° plane as an example. As shown in Figure 19b, the metasurface prototype and the transmitting horn antenna were placed at opposite ends of a turntable. Since the turntable only supports horizontal rotation, to obtain the pattern on the *φ* = 45° plane, both the sample and the transmitting antenna were rotated by 45° with their centers aligned. The receiving antenna was fixed in the far-field region, also rotated by 45°, and kept at the same height as the prototype and the transmitting antenna, as shown in Figure 19c. The measurement for the *φ* = 135° plane was performed in the same manner by rotating the sample and antennas to the corresponding orientation. During the measurement, the turntable was rotated while the transmission coefficient $S_{21}$ from the transmitter to the receiver was recorded in real time using a vector network analyzer (VNA, Ceyear 3672C, Ceyear Technologies Co., Ltd., Qingdao, China) to map out the scattering far-field pattern of the metasurface.

**Figure 20:**
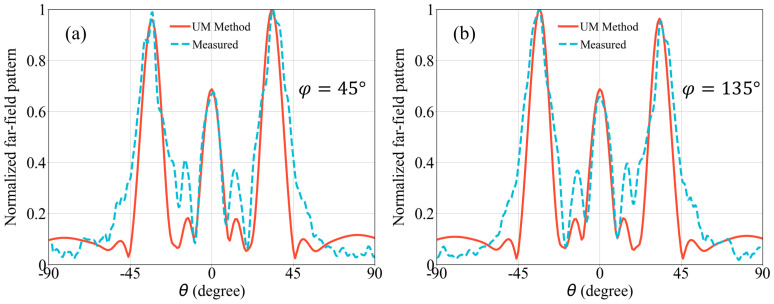
Normalized far-field patterns of the metasurface on the (**a**) *φ* = 45° and (**b**) φ = 135° planes.

Figure 20a,b present the normalized far-field patterns of the metasurface at 5 GHz on the *φ* = 45° and *φ* = 135° planes, respectively. Overall, the measured results follow the theoretical predictions of the UM method well, indicating that the metasurface exhibits five distinct scattering beams, including the central beam at *θ* = 0° and four surrounding beams located at *θ* = ±33° in the *φ* = 45° and *φ* = 135° planes. The central beam observed in the measured results, represented by the blue dashed lines, is accurately reproduced by the UM method, represented by the red solid lines. The parasitic sidelobes appearing around *θ* = ±14° are primarily caused by the reflected signal being blocked by the transmitting antenna. Furthermore, slight deviations and broadening are observed in the scattering beams at *θ* = ±33°. These discrepancies can be attributed to non-ideal multipath interference introduced by the anechoic chamber environment, the turntable, and the antenna mounts, as well as fabrication tolerances of the PCB. Despite these minor differences, the overall trends of the measured and calculated results are highly consistent. This fully demonstrates the effectiveness of the UM method in rapidly predicting the far-field scattering characteristics of coding metasurfaces, providing strong support for its applications in EM scattering manipulation.

## 5. Conclusions

This paper proposes a UM method to rapidly analyze the far-field scattering characteristics of coding metasurfaces. This method innovatively improves the equivalent source extraction strategy by comprehensively considering both the coupling effects and the positional influences of the metasurface elements. Comparisons against the conventional RPM method, ELP method, and full-wave simulation verify the comprehensive advantages of the proposed UM method in computational efficiency and accuracy. In addition, the experimental results further validate its reliability. The study demonstrates that the UM method can significantly reduce computation time while ensuring evaluation accuracy. It provides an efficient tool for fast far-field performance analysis of coding metasurfaces and lays a solid foundation for their intelligent design, showing broad application prospects in the field of EM scattering manipulation.

## Figures and Tables

**Table 1:** Regions of each element in the metasurface.

Region of the Element in the Metasurface	Element Numbering
Interior region	7, 8, 9, 12, 13, 14, 17, 18, 19
Top edge regions	2, 3, 4
Left edge regions	6, 11, 16
Right edge regions	10, 15, 20
Bottom edge regions	22, 23, 24
Top-left corner regions	1
Top-right corner regions	5
Bottom-left corner regions	21
Bottom-right corner regions	25

**Table 2:** Summary of the accuracy and computational time of different methods for the chessboard metasurface.

	Pearson Correlation Coefficient	RMSE	Computational Time
Full-wave simulation	\	\	4.5 h
UM method	0.991	0.060	80 s
ELP method	0.947	0.105	24 s
RPM method	0.893	0.145	1.6 s

**Table 3:** Summary of the accuracy and computational time of different methods for the beam-steering metasurface.

	Pearson Correlation Coefficient	RMSE	Computational Time
Full-wave simulation	\	\	1.2 h
UM method	0.986	0.052	36 s
ELP method	0.814	0.173	11 s
RPM method	0.955	0.086	0.6 s

## Data Availability

The original contributions presented in this study are included in the article/Supplementary Material. Further inquiries can be directed to the corresponding author.

## Supplementary Materials

The following supporting information can be downloaded at: https://www.mdpi.com/article/10.3390/ma19153203/s1, Supplementary Material S1: Robustness of Sampling Parameters to Element Structure, including Figure S1: (a) Schematic of the sub-array and the sampling plane; electric field intensity distributions at (b) z = λ/5, (c) z = λ/4, and (d) z = λ/3. Here, *p* denotes the element period; Figure S2: Variation rate of the $E_{x}$ component along the *x*-direction at *y = 3p*/*2* for different sampling distances; Figure S3: Comparison between equivalent source calculation results and full-wave simulation results under different sampling step sizes: (a) *φ* = 0°; (b) *φ* = 90°; (c) relative error at *φ* = 0°; (d) relative error at *φ* = 90°. Supplementary Material S2: Effect of Sub-Array Expansion on Prediction Accuracy and Computational Efficiency, including Figure S4: Schematic of the 7 × 7 metasurface (numbers 1 to 49 represent element numbering); Figure S5: Schematic of the equivalent source extraction models for elements in different regions of the metasurface: (a) top-left corner; (b) top edge; (c) top-right corner; (d) left edge; (e) interior; (f) right edge; (g) bottom-left corner; (h) bottom edge; (i) bottom-right corner; Figure S6: Normalized far-field patterns of the metasurface in the *φ* = 45° plane.
