# Supplementary material for: Fast Analysis Method for Far-Field Scattering Characteristics of Coding Metasurfaces Based on the Equivalent Source Principle
Source: Materials (Basel). 2026 Jul 27;19(15):3203. doi: 10.3390/ma19153203 (PMC13467058; doi:10.3390/ma19153203)
Supplement: Supplementary file 1 [file materials-19-03203-s001.zip › materials-4406987-supplementary.pdf]

Supporting Information

Fast Analysis Method for Far-Field Scattering Characteristics of Coding Metasurfaces Based on the Equivalent Source Principle

Rui Yang [1], Hanzhang Tao [2], Ge Fan [1], Junyan Dai [1,*] and Qiang Cheng [1]

S1: Robustness of Sampling Parameters to Element Structure

To verify the applicability of the parameter settings $t \geq \lambda/4$, $d \geq \lambda/4$, and sampling step $l \leq \lambda/10$ to different element structures, a further simulation analysis was conducted on a 3×3 sub-array composed of the "0" and "1" Jerusalem-cross elements proposed in Section 3.2. Under normal incidence of an x-polarized plane wave at 15 GHz, the electric field intensity distributions at different distances above the sub-array were calculated, as shown in Figure S1(a).

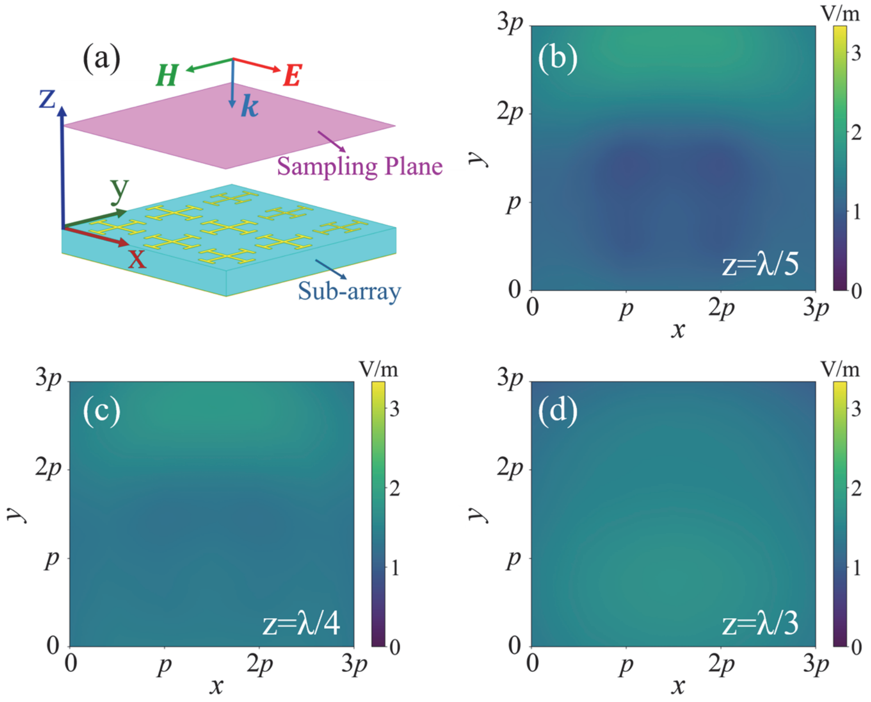

Figure S1. (a) Schematic of the sub-array and the sampling plane; electric field intensity distributions at (b) $z = \lambda/5$, (c) $z = \lambda/4$, and (d) $z = \lambda/3$. Here, p denotes the element period.

Figures S1(b)–(d) show the electric field intensity distributions on the sampling planes at $z = \lambda/5$, $z = \lambda/4$, and $z = \lambda/3$, respectively. As shown, the electric-field distribution gradually becomes

smoother as the sampling distance increases. To further quantify the spatial variation of the electric field, the variation rate of the E_x component along the *x*-direction was extracted at *y* = *3p/2* on each sampling plane, as shown in Figure S2. When the sampling distance is $\lambda/5$, the maximum variation rate is 93.5. As the sampling distance increases to $\lambda/4$, it decreases to 43.2. However, when the distance is further increased to $\lambda/3$, it no longer decreases and even slightly increases. These results indicate that setting the sampling distance to $\lambda/4$ or larger is appropriate, as it can effectively avoid the risk of undersampling caused by strong electromagnetic fluctuations.

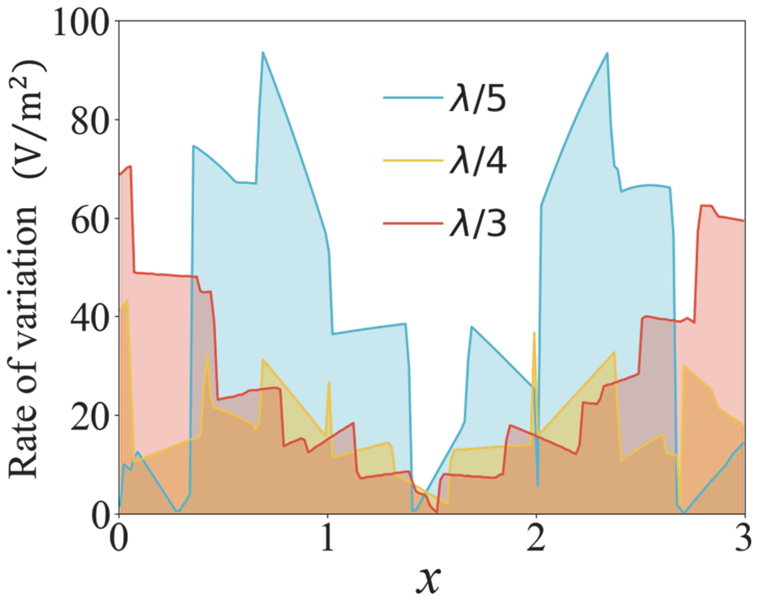

Figure S2. Variation rate of the E_x component along the *x*-direction at *y* = *3p/2* for different sampling distances.

With the sampling surface enclosing the sub-array and maintaining an appropriate distance from its surface, the influence of the sampling step *l* on the scattering far-field calculation results was further investigated. Figures S3(a) and S3(b) compare the far-fields calculated from the equivalent sources with the full-wave simulation results under different sampling steps in the $\varphi = 0°$ and $\varphi = 90°$ planes, respectively. Figures S3(c) and S3(d) show the corresponding relative errors.

As shown in the figures, the results calculated from the equivalent sources gradually approach the full-wave simulation results as the sampling step decreases. When the sampling step is reduced to $\lambda/10$, the maximum relative error decreases to approximately 15%. Although this value is still relatively large, it is mainly caused by the intrinsically low scattering-field amplitude in the corresponding direction, as shown in Figure S3(b). In contrast, in the main-beam directions, such as $\theta = 0°$ and $\varphi = 0°$, $\theta = 37°$, the relative errors are less than 5%. Therefore, setting the sampling step to $\lambda/10$ or smaller is reasonable.

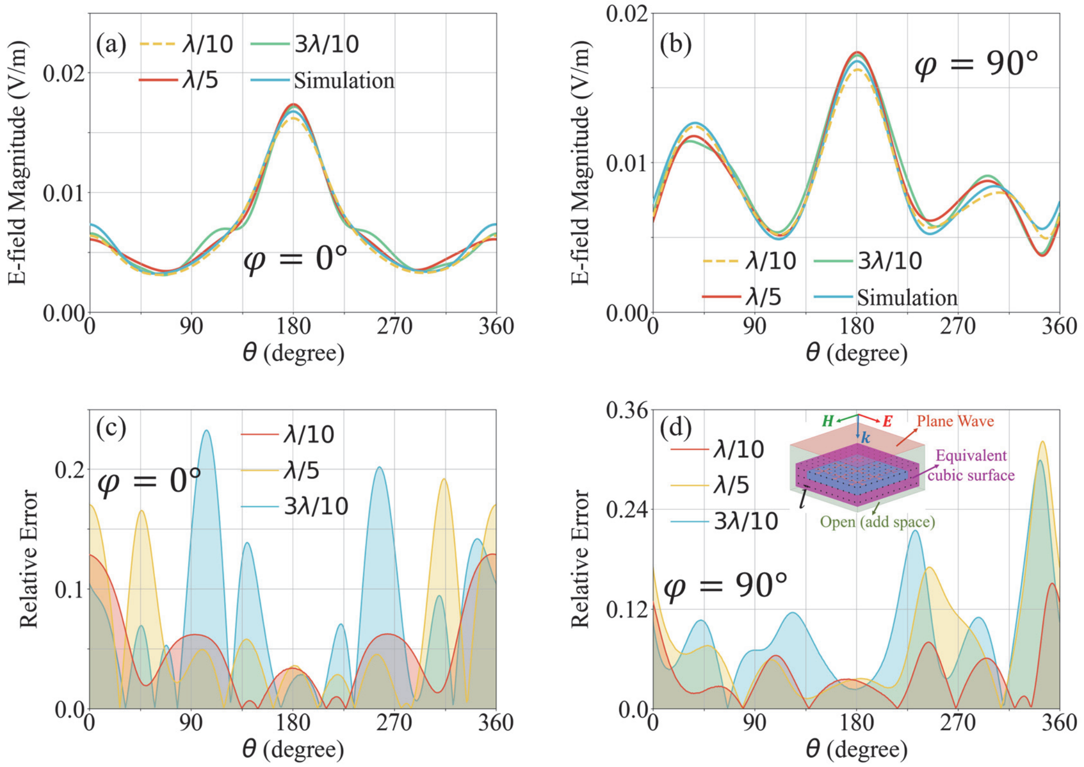

Figure S3. Comparison between equivalent source calculation results and full-wave simulation results under different sampling step sizes: (a) $\varphi = 0°$; (b) $\varphi = 90°$; (c) relative error at $\varphi = 0°$; (d) relative error at $\varphi = 90°$.

In summary, the parameter settings of a sampling distance no smaller than $\lambda/4$ and a sampling step no larger than $\lambda/10$ are applicable not only to arrays composed of square-ring elements but also to arrays composed of other types of elements. This indicates that the parameter settings are insensitive to the element structure and exhibit good generality.

S2: Effect of Sub-Array Expansion on Prediction Accuracy and Computational Efficiency

In this section, the sub-array model used for equivalent source extraction is extended to 5×5, so that the coupling effects from more neighboring elements can be taken into account while increasing the sub-array size. Taking the 7×7 array shown in Figure S4 as an example, the extraction strategy for elements at different positions is illustrated based on the 5×5 sub-array model. As shown in Figure S5, the extraction models for different element positions are similar to those in the 3×3 case, except that the 5×5 model further includes outer neighboring elements.

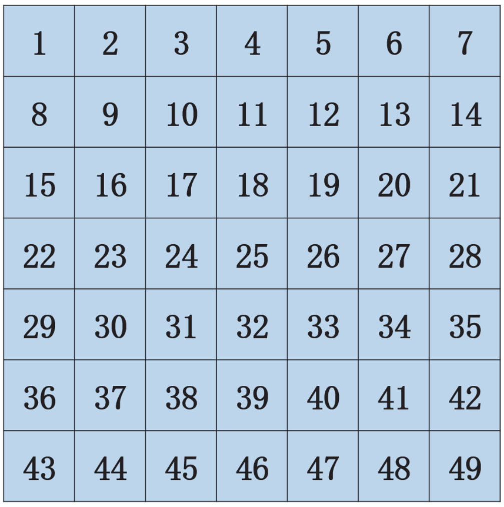

Figure S4. Schematic of the 7×7 metasurface (numbers 1 to 49 represent element numbering).

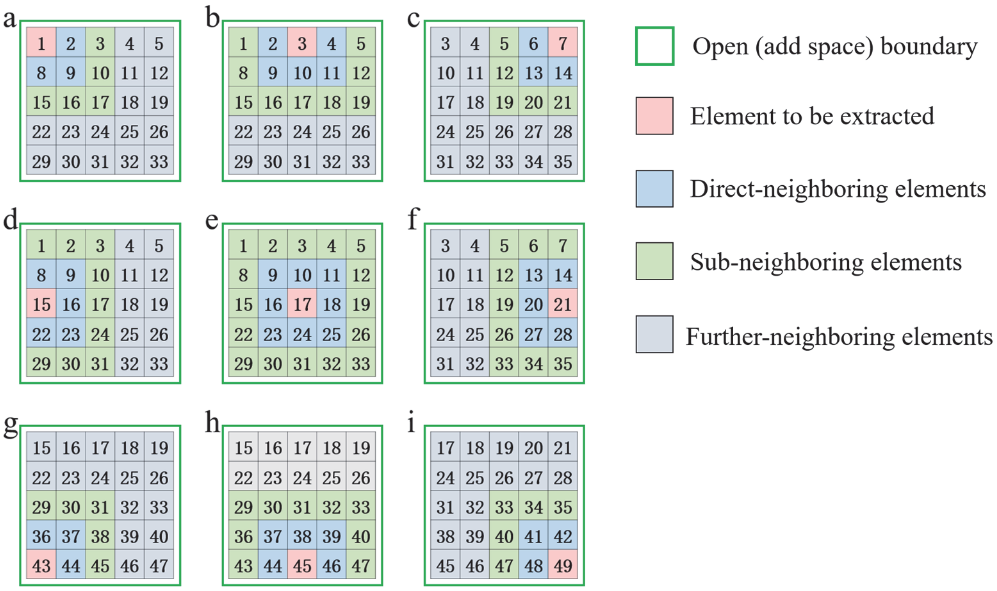

Figure S5. Schematic of the equivalent source extraction models for elements in different regions of the metasurface: (a) top-left corner; (b) top edge; (c) top-right corner; (d) left edge; (e) interior; (f) right edge; (g) bottom-left corner; (h) bottom edge; (i) bottom-right corner.

Furthermore, the 5×5 sub-array model is used to extract the equivalent sources, and the UM method is then employed to calculate the scattering far field of the chessboard metasurface shown in Figure 11 of Section 3.1. The corresponding result is plotted as the black dashed line in Figure S6. Compared with the 3×3 result, represented by the red dashed line, the 5×5 result agrees better with the full-wave simulation, represented by the yellow solid line, especially in the sidelobe regions. The Pearson correlation coefficient and RMSE reach 0.998 and 0.016, respectively. However, the improvement in the main-beam directions is limited, since the 3×3 case already provides accurate predictions in these directions.

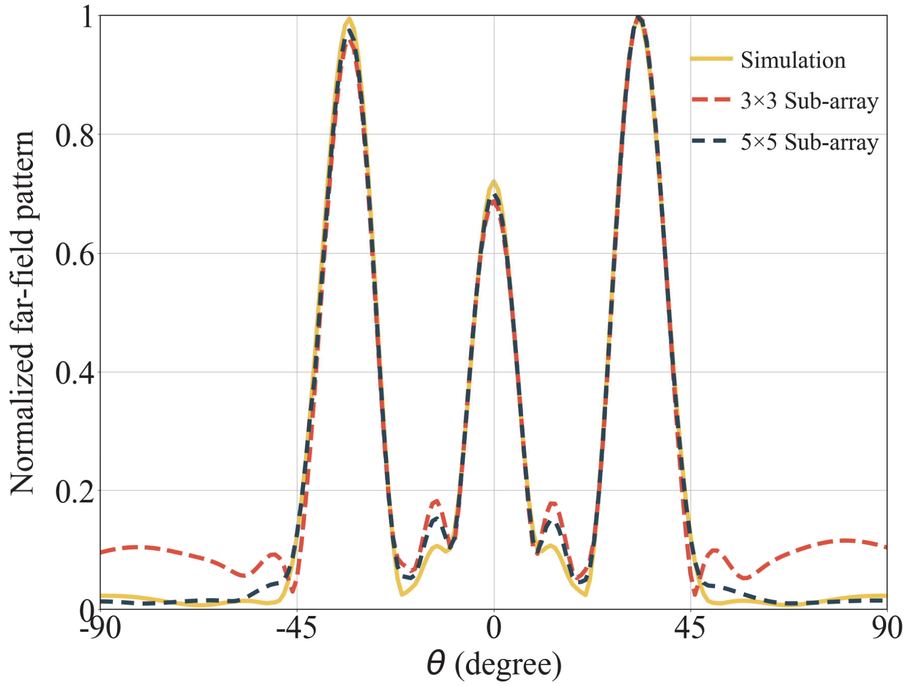

Figure S6. Normalized far-field patterns of the metasurface in the φ = 45° plane.

Nevertheless, the enlarged sub-array also increases the computational burden. The number of sub-array coding patterns increases from 18 to 50, and the simulation time for a single sub-array model increases from approximately 2.6 minutes to 3.2 minutes. Consequently, the total time required for equivalent-source extraction increases from approximately 47 minutes to about 2.7 hours. Considering both the improvement in accuracy and the increased computational cost, the 3×3 sub-array model is adopted as a more reasonable trade-off unless extremely high prediction accuracy is required.
